# Better adherence to childhood *Haemophilus influenzae* type b vaccination with combination vaccines compared to single-antigen vaccines: Evidence from China

**DOI:** 10.7189/jogh.13.04080

**Published:** 2023-08-25

**Authors:** Xiaozhen Lai, Yidi Ma, Hai Fang

**Affiliations:** 1 Department of Health Policy and Management, School of Public Health, Peking University, Beijing, China; 2 Health Economics Research Centre, Nuffield Department of Population Health, University of Oxford, Oxford, UK; 3 China Center for Health Development Studies, Peking University, Beijing, China; 4 Peking University Health Science Center-Chinese Center for Disease Control and Prevention Joint Research Center for Vaccine Economics, Peking University, Beijing, China

## Abstract

**Background:**

The coverage of *Haemophilus influenzae* type b (Hib) vaccination remains suboptimal in China, and this study aimed to investigate the influencing factors of caregivers’ Hib-containing vaccine choices and the association between combination vaccine use and adherence to Hib immunisation schedule among Chinese children.

**Methods:**

From August to October 2019, a cross-sectional survey was conducted in 148 community health care centres from ten provinces in China, which collected vaccination records from 5294 children aged 6-59 months. The children were categorised into three groups based on their Hib-containing vaccine receipt: unvaccinated group, monovalent vaccine group, and combination vaccine group. The outcome measures included: (1) receipt and choice of Hib-containing vaccines, and (2) completion of the three-dose schedule. Multinomial logistic regression was used to evaluate the influencing factors of Hib-containing vaccine receipt and choice, and logistic regression was adopted to investigate the associations between vaccine choice and schedule completion.

**Results:**

Of the 5294 children, 19.53% received monovalent vaccines only, 22.59% received at least one dose of combination vaccines, and 57.88% were not vaccinated against Hib. The overall three-dose completion rate was 27.03%. The multinomial logistic (mlogit) regression findings indicated strong associations of socioeconomic status and Hib-containing vaccine supply with vaccination status. Multiple logistic regression among those who received at least one Hib-containing dose demonstrated a 3-fold increase in the likelihood of three-dose schedule completion by children who received any combination dose compared with those received single-antigen vaccines only (adjusted odds ratio (aOR) = 3.97 (95% CI = 3.14-5.03)).

**Conclusions:**

Findings from the current study provide a more comprehensive understanding of the strong relationship between combination vaccine receipt and completion outcomes. Facing the suboptimal Hib vaccination rate in China, the use of combination vaccines could help increase vaccination compliance, and appropriate government actions to reduce out-of-pocket burden of immunisation are encouraged to address inequities in vaccine choices.

*Haemophilus influenzae* type b (Hib) is a prevalent cause of pneumonia, meningitis and other severe infections among children [1,2] and vaccination is considered as one of the most cost-effective public health measures to prevent Hib disease. The global incidence of Hib-related disease has exhibited a notable decline following the introduction of Hib vaccines [3,4] which have been incorporated into the National Immunization Programs (NIP) of 193 out of 194 World Health Organization (WHO) member countries and regions. However, China stands as the sole WHO member that has yet to include Hib vaccine in its NIP, despite its large remaining Hib disease burden [5]. China was estimated to be among the ten countries with the greatest number of Hib cases and deaths in children aged 1-59 months in 2000 [6] and an estimated 2900 Hib-related deaths persisted in China in 2017 [5].

Receiving recommended childhood vaccinations on schedule is a cornerstone of public health, while insufficient vaccination coverage and poor adherence to vaccine schedules can engender an elevated risk of disease outbreaks. In the past decades, the introduction of vaccination initiatives in China has resulted in an effective rise in NIP vaccine coverage to over 95%, but the coverage of non-NIP vaccines such as Hib vaccine remains suboptimal [7]. Hib vaccine was made available in China’s private sector in 1996, and a uniform policy was implemented across different regions regarding its administration, under which children and parents who are willing to bear the expenses are granted consistent access to the vaccine. Given the out-of-pocket cost of Hib vaccination, especially more expensive Hib-containing combination vaccines, the overall coverage rate of Hib vaccination was approximately 30%, implying a substantial portion of unvaccinated population [5]. The Chinese Center for Disease Control and Prevention (CDC) recommends administering Hib vaccines at the third, fourth and fifth months, with a booster dose at half past one year. However, between the ages of two and six months, a series of standard antigens were recommended by the Chinese CDC, including NIP vaccines like Bacillus Calmette-Guérin vaccine (BCG), a combination vaccine against diphtheria, tetanus and pertussis (DTaP), inactivated polio vaccine (IPV), measles vaccine, etc., as well as non-NIP vaccines against Hib, pneumococcus, rotavirus, and rubella. Given the increasing number of recommended vaccines, completing all NIP schedules for children under six months old requires around eleven injections and up to seven visits.

Vaccination at age-appropriate intervals increases protection against morbidity and mortality. However, compliance rates among children remain low partly due to the complexity of the vaccination schedule. Previous studies have confirmed that immunisation rates cannot be solely improved by increasing the number of injections during one visit [8]. Although giving simultaneous injections is considered safe and effective in guidelines [9], the increased number of injections received per visit and the complicated timing of each dose in the recommended schedule can be burdensome for parents [10], which was reported to be the second most common reason for parental resistance and lack of compliance with the vaccination schedule [11]. By grouping several antigens into one injection, combination vaccines, represent an effective way of increasing vaccination rates [12]. With valid clinical evidence suggesting good immunogenicity and safety, various Hib-containing combination vaccines are currently available, and are featured in the majority of developed countries’ vaccination schedules to effectively reduce the number of required injections and visits [13,14].

Compared with single-antigen formulations, presumed advantages of combination vaccines have been demonstrated in terms of benefits to individuals, the society and health care system [15]. The evolution of Hib-containing vaccines from monovalent to different combination formulations allows for fewer injections and patient visits, potentially lower non-medical and indirect costs, higher efficiency of health care practice, and thus improved vaccine coverage rates and compliance with the vaccination schedule [15,16]. Studies also have confirmed that the effectiveness of combination vaccines remains uncompromised [17]. In China, there are four Hib-containing vaccines available for use by vaccine providers (**Table 1**), including monovalent, trivalent, quadrivalent and pentavalent vaccines, each with different approved dosage schedules. The combination vaccines combine non-NIP Hib antigen with NIP vaccines such as DTaP vaccine, IPV and meningococcal vaccine. More specifically, monovalent Hib vaccine is indicated for active immunisation against Hib, trivalent vaccine is against group A and C meningococcal infections and Hib, quadrivalent vaccine is against diphtheria, tetanus, pertussis and Hib, and pentavalent vaccine adds polio virus to the antigen list compared with quadrivalent vaccines. The general age indication for the four-dose monovalent, quadrivalent and pentavalent Hib-containing vaccines is ≥6 weeks or <2nd birthday, except for the AC group meningococcal (AC)-Hib vaccine, which only requires one to three doses based on the child’s age.

**Table 1:** *Haemophilus influenzae* type b (Hib)-containing vaccines available during the study period from 2014 to 2019

Formulation	Valent	Approved doses	Price (yuan)*
Hib	Monovalent	4 doses (3 + 1)	62-100.8
AC-Hib	Trivalent	3 doses for children aged 2-5 mo; 2 doses for children aged 6-11 mo; 1 dose for children aged 12-71 mo	210-273
DTaP / Hib	Quadrivalent	4 doses (3 + 1)	275
DTaP-IPV / Hib	Pentavalent	4 doses (3 + 1)	599

Hib – *Haemophilus influenzae* type b, AC – AC group meningococcal, DTaP – diphtheria, tetanus and pertussis, IPV – inactivated polio vaccine, mo – months

*Vaccine prices are obtained from the centralised procurement list of non-NIP vaccines in China in 2018.

Previous studies have provided valuable insights into the association between combination vaccine use and vaccination behaviours. Research conducted in the United States often focused on DTaP-containing [12,18-20] or hepatitis B [12,21] combination vaccines, demonstrating that children receiving combination vaccines had higher completion rates for the recommended vaccination series. However, to our knowledge, there is still a lack of evidence quantifying the effects of combination vaccines in China, the world’s most populous country, and the attention given to Hib-containing combination vaccines is limited. Facing the suboptimal Hib vaccination rate but high NIP vaccination coverage, studying the effects of Hib-containing combination vaccines will help bridge critical knowledge gaps. Therefore, this study aims to investigate influencing factors of caregivers’ Hib-containing vaccine type choices and the association between combination vaccine use and adherence to the recommended Hib immunisation schedule among Chinese children, using data from a large-scale nationwide survey with childhood vaccination records.

Based on the insights above, this study proposed two hypotheses. H1: more socioeconomically deprived children were less likely to receive Hib-containing vaccination, especially more-expensive combination vaccines. H2: combination vaccine recipients were more likely to complete the three-dose schedule than monovalent vaccine recipients.

## METHODS

### Study design

From August to October 2019, a cross-sectional survey was conducted in 148 community health care centres across ten provinces in China, which also collected vaccination records of children aged 6-59 months until the time of the survey. The survey utilised a multistage cluster sampling method to select community health care centres from ten provinces / provincial-level cities in China [22]. The selected community health care centres were primary health care institutions in China which provide basic health services to local residents, including public health services (vaccination, health education, etc.) and medical services (common disease treatment, traditional Chinese medicine, etc.) [23]. In China, all children under the age of five are expected to receive NIP vaccines based on the routine vaccination schedule at these vaccination centres. Therefore, the participants enrolled in the on-site survey can be considered as a representative sample of local children, as the NIP vaccine coverage in China was approximately 99% [24]. The study was ethically reviewed and approved by the Peking University Institutional Review Board (IRB00001052-19076), and written informed consent was obtained from caregivers.

The questionnaires were administered face-to-face by trained interviewers using a specially designed online system on portable android device (PAD) based on the caregivers’ (parents or grandparents) stated answers, which allowed quality control in a timely manner. The survey questionnaire included information on the socio-demographic characteristics of the children (e.g., child’s age, gender, and number of children in a family) and their caregivers (e.g., guardian’s age, relationship with the child, ethnicity, education level, household income, status of residence, and place of residence). The PAD was also used to capture snapshots of children’s vaccination records, including the type and date of different vaccine doses received by each child, were clearly written or printed to avoid recall bias in self-reported vaccination status. The sample size of the study was set at 3840 participants (**Online Supplementary Document**), and was increased in practice to accommodate the response rate and ensure data integrity. In practice, 6668 children were recruited in the survey. Among the sample of 6668 children, the caregivers of 5384 (80.74%) children agreed to provide their vaccination records, and records of 5294 (79.39%) children were legible and complete with snapshots for every page.

### Study measures

Children were categorised into three mutually exclusive groups based on their receipt of Hib-containing vaccines, including the unvaccinated group who were not vaccinated against Hib, the monovalent vaccine group who received single-antigen vaccines only, and the combination vaccine group who received at least one dose of combination vaccine (i.e., combination vaccines only, or a mixture of combination and single-antigen vaccines). The outcome measures included: (1) receipt and choice of Hib-containing vaccines, and (2) completion of the three-dose schedule. The former measured Hib vaccination coverage stratified by Hib-containing vaccine use, while the latter assessed the completion of the three-dose schedule among children who received at least one dose of Hib-containing vaccines. In China, the first three Hib-containing vaccine doses are recommended to be administered at two-five months. Given that AC-Hib is only administered for three doses while others were approved for four doses, vaccine completion was defined as receiving the required three doses of Hib-containing vaccines by a specific age (5 + 1 month) regardless of the timing of vaccine administration.

To explore the effects of potential confounding factors other than Hib-containing vaccine type, the outcomes were adjusted for sociodemographic characteristics (respondent's age, respondent's relationship with the child, respondent's education level, minority group status, child's age, child's gender, household monthly per capita income, place of residence, migrant status, province), vaccine confidence indicators (considering vaccine as important, safe or effective), and vaccine availability (whether the frequently visited health care centre provided Hib monovalent vaccine and combination vaccine in the past year). The questions related to vaccine availability were answered by the directors of vaccination sites in health care centres to supplement the information that could not be obtained directly from individual respondents.

### Statistical analysis

Frequencies and proportions were used to describe the characteristics of children and their caregivers for the overall population and stratified by Hib vaccination type. The χ^2^ test was used to assess differences in sample characteristics. Multinomial logistic regression was used to evaluate the influencing factors of Hib-containing vaccine receipt and choice, taking the unvaccinated group as the reference. The regression analysis was adjusted for the characteristics at caregiver, child and household levels, vaccine hesitancy measures, and vaccine availability at the facility level. Later on, logistic regression was adopted to investigate the associations between vaccine choice and schedule completion among those who received at least one Hib-containing dose, which was also controlled for the above factors. Relative-risk ratio (RRR), adjusted odds ratio (aOR), and their 95% confidence intervals (CIs) were reported. A two-sided *P*-value below 0.05 was considered statistically significant in the present study. The data were analysed using Stata version 14.0 (Stata Corp., College Station, TX, USA).

## RESULTS

### Study sample characteristics

**Table 2** shows the socio-demographic characteristics of 5294 respondents and their children with complete and legible vaccination records. Of the children, 19.53% received monovalent vaccines only, 22.59% received at least one dose of combination vaccines, and 57.88% were not vaccinated against Hib. The majority of sampled caregivers were aged less than 40 years (77.33%), mothers (66.75%), educated beyond high school (64.56%), and not belonging to ethnic minority groups (94.14%). More children were male (52.81%), resided in urban areas (59.60%) and were not inter-city migrants (76.07%). As for vaccine-related attitudes, most caregivers perceived high importance (97.45%), safety (80.85%) and efficacy (80.13%) of vaccination. In frequently visited health care centres, Hib vaccines were available for the majority of children (monovalent 84.74%; combination 61.58%), and the overall three-dose completion rate was 27.03%.

**Table 2:** Basic characteristics of respondents by Hib vaccination status

	Overall		Unvaccinated group		Monovalent vaccine group		Combination vaccine group		*P*-value*
	**N**	**Column %**	**N**	**Column %**	**N**	**Column %**	**N**	**Column %**
**Total**	5294	100.00	3064	100.00	1034	100.00	1196	100.00	
**Respondent's age (years)**									0.025
<30	1637	30.92	963	31.43	323	31.24	351	29.35	
30-39	2457	46.41	1430	46.67	442	42.75	585	48.91	
40-49	458	8.65	244	7.96	107	10.35	107	8.95	
≥50	742	14.02	427	13.94	162	15.67	153	12.79	
**Respondent's relationship with the child**									0.009
Mather	3534	66.75	2011	65.63	696	67.31	827	69.15	
Father	907	17.13	558	18.21	150	14.51	199	16.64	
Grandparent	853	16.11	495	16.16	188	18.18	170	14.21	
**Respondent's education level**									<0.001
Elementary school and below	521	9.84	317	10.35	153	14.80	51	4.26	
Middle school	1355	25.60	821	26.80	342	33.08	192	16.05	
Senior high school / technical school	1194	22.55	737	24.05	232	22.44	225	18.81	
College / associate degree	997	18.83	565	18.44	161	15.57	271	22.66	
Bachelor's degree and above	1227	23.18	624	20.37	146	14.12	457	38.21	
**Belonging to minority groups**									<0.001
Yes	310	5.86	175	5.71	89	8.61	46	3.85	
No	4984	94.14	2889	94.29	945	91.39	1150	96.15	
**Child's age (years)**									<0.001
<1	1439	27.18	879	28.69	140	13.54	420	35.12	
1-2	1547	29.22	909	29.67	276	26.69	362	30.27	
2-3	981	18.53	564	18.41	221	21.37	196	16.39	
3-5	1327	25.07	712	23.24	397	38.39	218	18.23	
**Child's gender**									0.248
Female	2498	47.19	1416	46.21	504	48.74	578	48.33	
Male	2796	52.81	1648	53.79	530	51.26	618	51.67	
**Household monthly per capita income quantiles**									<0.001
Quantile 1 (0-1000 yuan)	1152	21.76	774	25.26	259	25.05	119	9.95	
Quantile 2 (1001-1600 yuan)	900	17.00	552	18.02	201	19.44	147	12.29	
Quantile 3 (1601-2400 yuan)	1063	20.08	627	20.46	214	20.70	222	18.56	
Quantile 4 (2401-3750 yuan)	1132	21.38	616	20.10	197	19.05	319	26.67	
Quantile 5 (>3751 yuan)	1047	19.78	495	16.16	163	15.76	389	32.53	
**Place of residence**									<0.001
Rural	2139	40.40	1323	43.18	518	50.10	298	24.92	
Urban	3155	59.60	1741	56.82	516	49.90	898	75.08	
**Migrant respondent**									0.501
Yes	1267	23.93	744	24.28	233	22.53	290	24.25	
No	4027	76.07	2320	75.72	801	77.47	906	75.75	
**Consider vaccine as important**									0.399
Yes	5159	97.45	2981	97.29	1006	97.29	1172	97.99	
No	135	2.55	83	2.71	28	2.71	24	2.01	
**Consider vaccine as safe**									0.784
Yes	4280	80.85	2486	81.14	829	80.17	965	80.69	
No	1014	19.15	578	18.86	205	19.83	231	19.31	
**Consider vaccine as effective**									0.150
Yes	4242	80.13	2483	81.04	817	79.01	942	78.76	
No	1052	19.87	581	18.96	217	20.99	254	21.24	
**Hib monovalent vaccine provided in frequently visited health care centre**									<0.001
Yes	4486	84.74	2299	75.03	1026	99.23	1161	97.07	
No	808	15.26	765	24.97	8	0.77	35	2.93	
**Hib combination vaccine provided in frequently visited health care centre**									<0.001
Yes	3260	61.58	1694	55.29	547	52.90	1019	85.20	
No	2034	38.42	1370	44.71	487	47.10	177	14.80	
**Three-dose Hib-containing vaccine immunisation completion**									<0.001
Yes	1431	27.03	0	0.00	491	47.49	940	78.60	
No	3863	72.97	3064	100.00	543	52.51	256	21.40	
**Province**									<0.001
Shanghai	525	9.92	155	5.06	99	9.57	271	22.66	
Beijing	551	10.41	490	15.99	18	1.74	43	3.60	
Chongqing	567	10.71	291	9.50	179	17.31	97	8.11	
Gansu	390	7.37	316	10.31	21	2.03	53	4.43	
Guangdong	511	9.65	308	10.05	102	9.86	101	8.44	
Henan	516	9.75	172	5.61	111	10.74	233	19.48	
Jiangxi	554	10.46	229	7.47	188	18.18	137	11.45	
Jilin	613	11.58	541	17.66	34	3.29	38	3.18	
Shandong	522	9.86	309	10.08	93	8.99	120	10.03	
Yunnan	545	10.29	253	8.26	189	18.28	103	8.61	

Hib – *Haemophilus influenzae* type b

*P-values from χ^2^ test.

There were also significant demographic differences between the three groups in terms of caregivers’ characteristics including age (*P* = 0.025), relationship with the child (*P* = 0.009), education level (*P* < 0.001) and minority group identity (*P* < 0.001), as well as child’s age (*P* < 0.001), household per capita income (*P* < 0.001), and place of residence (*P* < 0.001). Moreover, there were between-group differences concerning the shortage of monovalent vaccines (*P* < 0.001) and combination vaccines (*P* < 0.001) in frequently visited health care centres, and the completion of three-dose Hib vaccination schedule (*P* < 0.001).

### Associated factors of Hib vaccination status

This study investigated the influencing factors of Hib vaccination status by multinomial logistic regressions, taking the unvaccinated group as the base (**Table 3**). It was found that children in households with higher quantiles of income were more like to vaccinate against monovalent Hib vaccines (quantile 3 RRR = 1.36 (95% CI = 1.06-1.74); quantile 4 RRR = 1.34 (95% CI = 1.03-1.75); quantile 5 RRR = 1.58 (95% CI = 1.17-2.13)) and combination vaccines (quantile 2 RRR = 1.45 (95% CI = 1.08-1.95); quantile 3 RRR = 1.72 (95% CI = 1.30-2.28); quantile 4 RRR = 1.94 (95% CI = 1.46-2.58); quantile 5 RRR = 2.65 (95% CI = 1.96-3.58)) compared with those in quantile 1. Differences also existed concerning the influencing factors of monovalent and combination vaccine receipt. Compared with caregivers with elementary school education and below, those with a bachelor's degree and above were less likely to vaccinate their children against monovalent Hib vaccines (RRR = 0.64 (95% CI = 0.44-0.92)), while education beyond middle school was positively associated with childhood combination vaccine receipt (middle school RRR = 1.66 (95% CI = 1.12-2.47); senior high school / technical school RRR = 1.61 (95% CI = 1.07-2.42); college / associate degree RRR = 2.50 (95% CI = 1.63-3.83); bachelor's degree and above RRR = 3.45 (95% CI = 2.24-5.30)). Besides, compared with young children aged under one-year-old, those of older age were more like to receive monovalent vaccines (one-two years RRR = 2.09 (95% CI = 1.65-2.66); two-three years RRR = 2.53 (95% CI = 1.96-3.27); three-five years RRR = 3.36 (95% CI = 2.66-4.26)), while less likely to receive combination ones (two-three years RRR = 0.71 (95% CI = 0.56-0.90); three-five years RRR = 0.54 (95% CI = 0.43-0.67)). Caregivers aged 40-49 years (RRR = 1.54 (95% CI = 1.10-2.16)), urban residence (RRR = 1.35 (95% CI = 1.10-1.65)) and migrant status (RRR = 0.70 (95% CI = 0.58-0.86)) were found to be associated with combination vaccine receipt, but such relationships were not observed for monovalent Hib vaccination.

**Table 3:** Multinomial logistic regression to identify influencing factors of *Haemophilus influenzae* type b (Hib) vaccination status, taking unvaccinated group as the base

Factors	Monovalent vaccine group		Combination vaccine group	
	**RRR**	**95% CI**	**RRR**	**95% CI**
**Respondent's age (years)**				
<30	Ref.		Ref.	
30-39	0.97	(0.80-1.17)	0.96	(0.80-1.16)
40-49	1.20	(0.86-1.67)	1.54*	(1.10-2.16)
≥50	1.24	(0.66-2.32)	1.44	(0.71-2.90)
**Respondent's relationship with the child**				
Mather	Ref.		Ref.	
Father	0.84	(0.67-1.05)	0.85	(0.69-1.06)
Grandparent	0.72	(0.40-1.30)	1.17	(0.60-2.30)
**Respondent's education level**				
Elementary school and below	Ref.		Ref.	
Middle school	1.04	(0.77-1.39)	1.66*	(1.12-2.47)
Senior high school / technical school	0.78	(0.57-1.07)	1.61*	(1.07-2.42)
College / associate degree	0.71	(0.50-1.02)	2.50†	(1.63-3.83)
Bachelor's degree and above	0.64*	(0.44-0.92)	3.45†	(2.24-5.30)
**Belonging to minority groups**	1.11	(0.80-1.54)	0.70	(0.47-1.04)
**Child's age (years)**				
<1	Ref.		Ref.	
1-2	2.09†	(1.65-2.66)	0.96	(0.79-1.18)
2-3	2.53†	(1.96-3.27)	0.71†	(0.56-0.90)
3-5	3.36†	(2.66-4.26)	0.54†	(0.43-0.67)
**Child's gender is male**	0.88	(0.75-1.03)	0.90	(0.77-1.05)
**Household monthly per capita income quantiles**				
Quantile 1 (0-1000 yuan)	Ref.		Ref.	
Quantile 2 (1001-1600 yuan)	1.22	(0.96-1.56)	1.45*	(1.08-1.95)
Quantile 3 (1601-2400 yuan)	1.36*	(1.06-1.74)	1.72†	(1.30-2.28)
Quantile 4 (2401-3750 yuan)	1.34*	(1.03-1.75)	1.94†	(1.46-2.58)
Quantile 5 (>3751 yuan)	1.58†	(1.17-2.13)	2.65†	(1.96-3.58)
**Living in urban area**	0.86	(0.71-1.04)	1.35†	(1.10-1.65)
**Migrant respondent**	1.00	(0.81-1.23)	0.70†	(0.58-0.86)
**Consider vaccine as important**	1.08	(0.66-1.78)	0.90	(0.53-1.53)
**Consider vaccine as safe**	0.93	(0.75-1.16)	1.01	(0.82-1.26)
**Consider vaccine as effective**	1.00	(0.81-1.23)	0.83	(0.67-1.02)
**Hib monovalent vaccine provided in frequently visited health care centre**	22.11†	(10.45-46.77)	4.48†	(3.05-6.59)
**Hib combination vaccine provided in frequently visited health care centre**	0.93	(0.76-1.15)	2.38†	(1.89-2.99)
**Province**				
Shanghai	Ref.		Ref.	
Beijing	0.07†	(0.04-0.12)	0.05†	(0.03-0.07)
Chongqing	1.08	(0.75-1.54)	0.29†	(0.21-0.41)
Gansu	0.49*	(0.28-0.88)	0.36†	(0.23-0.55)
Guangdong	0.60*	(0.41-0.89)	0.41†	(0.29-0.58)
Henan	1.04	(0.71-1.52)	1.41*	(1.02-1.94)
Jiangxi	1.32	(0.93-1.89)	0.49†	(0.35-0.68)
Jilin	0.24†	(0.15-0.39)	0.14†	(0.09-0.21)
Shandong	0.67*	(0.45-0.99)	0.40†	(0.29-0.57)
Yunnan	1.12	(0.77-1.63)	0.62†	(0.43-0.88)

RRR – relative risk reduction, CI – confidence interval, Hib – *Haemophilus influenzae* type b

*P < 0.05.

†*P* < 0.01.

Vaccine supply in frequently visited health care centres was also related to vaccination status. Monovalent vaccine receipt had strong positive correlations with monovalent vaccine supply in health care centres (RRR = 22.11 (95% CI = 10.45-46.77)), while combination vaccine receipt was positively associated with the supply of both monovalent (RRR = 4.48 (95% CI = 3.05-6.59)) and combination (RRR = 2.38 (95% CI = 1.89-2.99)) vaccines. No significant association was found between vaccine confidence measures and Hib vaccination status.

### Associated factors of Hib vaccination completion

Multiple logistic regression adjusted for the type of received Hib vaccine, sociodemographic characteristics, attitudes towards vaccination and vaccine supply was performed to identify the influencing factors of three-dose Hib vaccination completion among those who received at least one Hib-containing dose, as shown in Model 1 in **Table 4**. The type of received Hib vaccine was a strong predictor of completion, where combination vaccine receipt was related to higher odds of completion (aOR = 3.96 (95% CI = 3.13-5.01)). For sociodemographic characteristics, some factors were observed to be associated with higher odds of completion, including caregivers with bachelor’s degree and above (aOR = 1.75 (95% CI = 1.07-2.87)), children aged three-five years (aOR = 1.76 (95% CI = 1.30-2.37)), higher quantiles of income (quantile 2 aOR = 1.66 (95% CI = 1.19-2.31); quantile 3 aOR = 1.45 (95% CI = 1.05-2.01); quantile 4 aOR = 1.83 (95% CI = 1.30-2.57); quantile 5 aOR = 2.33 (95% CI = 1.61-3.38)), and urban residence (aOR = 1.37 (95% CI = 1.06-1.78)). Vaccine confidence measures and institution vaccine supply were not found to be related to Hib vaccination completion.

**Table 4:** Logistic regression to identify influencing factors of *Haemophilus influenzae* type b (Hib) vaccination completion

Factors	Model 1		Model 2	
	**aOR**	**95% CI**	**aOR**	**95% CI**
**The type of received Hib vaccine**				
Monovalent vaccine only	Ref.		Ref.	
Combination vaccines	3.96†	(3.13-5.01)	-	-
AC-Hib only	-	-	1.16	(0.85-1.60)
DTaP-Hib only	-	-	6.01†	(4.15-8.70)
DTaP-IPV-Hib only	-	-	7.68†	(4.95-11.93)
Mixed vaccination	-	-	15.43†	(8.83-27.00)
**Respondent's age (years)**				
<30	Ref.		Ref.	
30-39	1.23	(0.97-1.56)	1.21	(0.95-1.55)
40-49	1.20	(0.80-1.80)	1.21	(0.79-1.84)
≥50	1.55	(0.69-3.50)	1.49	(0.66-3.40)
**Respondent's relationship with the child**				
Mather	Ref.		Ref.	
Father	0.86	(0.65-1.14)	0.88	(0.66-1.17)
Grandparent	1.18	(0.54-2.59)	1.10	(0.50-2.42)
**Respondent's education level**				
Elementary school and below	Ref.		Ref.	
Middle school	1.33	(0.88-2.02)	1.27	(0.83-1.93)
Senior high school / technical school	1.43	(0.92-2.22)	1.33	(0.84-2.08)
College / associate degree	1.42	(0.88-2.30)	1.30	(0.80-2.12)
Bachelor's degree and above	1.75*	(1.07-2.87)	1.55	(0.94-2.56)
**Belonging to minority groups**	0.95	(0.61-1.48)	1.01	(0.64-1.58)
**Child's age (years)**				
<1	Ref.		Ref.	
1-2	1.32	(1.00-1.74)	1.33	(1.00-1.78)
2-3	1.06	(0.78-1.44)	0.91	(0.66-1.26)
3-5	1.76†	(1.30-2.37)	1.54†	(1.13-2.11)
**Child's gender is male**	0.87	(0.71-1.06)	0.88	(0.71-1.07)
**Household monthly per capita income quantiles**				
Quantile 1 (0-1000 yuan)	Ref.		Ref.	
Quantile 2 (1001-1600 yuan)	1.66†	(1.19-2.31)	1.73†	(1.24-2.43)
Quantile 3 (1601-2400 yuan)	1.45*	(1.05-2.01)	1.51*	(1.08-2.10)
Quantile 4 (2401-3750 yuan)	1.83†	(1.30-2.57)	1.97†	(1.39-2.80)
Quantile 5 (>3751 yuan)	2.33†	(1.61-3.38)	2.20†	(1.50-3.24)
**Living in urban area**	1.37*	(1.06-1.78)	1.11	(0.85-1.45)
**Migrant respondent**	0.87	(0.67-1.13)	0.87	(0.66-1.14)
**Consider vaccine as important**	0.61	(0.30-1.20)	0.63	(0.31-1.26)
**Consider vaccine as safe**	0.88	(0.67-1.15)	0.85	(0.64-1.13)
**Consider vaccine as effective**	1.15	(0.89-1.50)	1.16	(0.89-1.52)
**Hib monovalent vaccine provided in frequently visited health care centre**	2.21	(0.93-5.25)	2.71*	(1.01-7.25)
**Hib combination vaccine provided in frequently visited health care centre**	1.14	(0.87-1.50)	1.15	(0.87-1.52)
**Province**				
Shanghai	Ref.		Ref.	
Beijing	0.22†	(0.12-0.43)	0.20†	(0.10-0.40)
Chongqing	0.86	(0.55-1.35)	0.97	(0.61-1.54)
Gansu	0.22†	(0.12-0.40)	0.19†	(0.10-0.37)
Guangdong	0.30†	(0.19-0.48)	0.33†	(0.20-0.53)
Henan	1.04	(0.67-1.60)	1.52	(0.96-2.41)
Jiangxi	0.86	(0.56-1.33)	0.96	(0.61-1.50)
Jilin	0.04†	(0.02-0.10)	0.09†	(0.04-0.22)
Shandong	0.48†	(0.30-0.76)	0.53*	(0.32-0.86)
Yunnan	0.66	(0.42-1.05)	0.81	(0.50-1.31)

aOR – adjusted odds ratio, CI – confidence interval, Hib – *Haemophilus influenzae* type b, AC – AC group meningococcal, DTaP – diphtheria, tetanus and pertussis, IPV – inactivated polio vaccine

*P < 0.05.

†*P* < 0.01.

**Figure 1:**
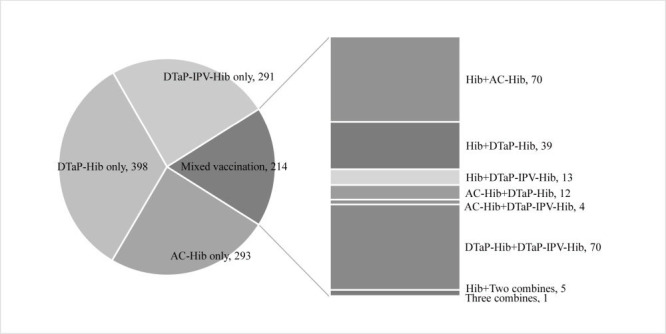
The composition of received Hib vaccine in the combination vaccine group (n = 1196). Hib – *Haemophilus influenzae* type b, AC – AC group meningococcal, DTaP – diphtheria, tetanus and pertussis, IPV – inactivated polio vaccine.

We then conducted multiple logistic regression where combination vaccination status was further divided into four subgroups, including AC-Hib only, DTaP-Hib only, DTaP-IPV-Hib only, and mixed vaccination, as shown in **Figure 1** and Model 2 in **Table 4**. In this way, the associations between Hib vaccination status and vaccination completion were refined to certain types of combination vaccines received by children after controlling for individual, household, and provider characteristics. It was found that except for the AC-Hib only group, other combination vaccine groups remained a strong predictor of higher odds of completion compared with those who received monovalent vaccine only, including DTaP-Hib only (aOR = 6.01 (95% CI = 4.15-8.70)), DTaP-IPV-Hib only (aOR = 7.68 (95% CI = 4.95-11.93)), and mixed vaccination (aOR = 15.43 (95% CI = 8.83-27.00)).

## DISCUSSION

This study is the first to assess caregivers’ Hib-containing vaccine choices and the association between combination vaccine use and schedule completion in China using real-world childhood vaccination records. The analysis revealed that approximately 40% of the surveyed children received at least one dose of Hib-containing vaccines. The regression findings showed strong associations of socioeconomic status and vaccine supply with caregivers’ Hib-containing vaccine choices, but also pronounced relationships between the receipt of combination vaccines and the successful completion of the vaccination schedule, supporting the previously proposed hypotheses H1 and H2.

Hib-containing combination vaccines (i.e., trivalent, quadrivalent and pentavalent) are presumed to improve vaccination timeliness, but research supporting this claim is limited. Previous studies on combination vaccines mainly focused on DTaP vaccination., and were not conducted in China. Findings from this study using multivariable logistic regression on three-dose completion indicated that children who received combination vaccines were nearly four times more likely to complete the three-dose schedule compared with monovalent vaccine recipients. This finding is consistent with those demonstrated in prior studies that DTaP-containing combination vaccine receipt was associated with significantly greater adherence to DTaP immunisation schedule [18,20,21]. For instance, a retrospective study of administrative Medicaid claims showed that children who received at least one dose of DTaP-HepB-IPV combination vaccine had a higher completion rate for the recommended vaccination series as well as higher rates of individual antigens [21]. Another study using data from the 2012 National Immunization Survey in the United States reported a more than a 2-fold increase in the likelihood of completing vaccination among children who received combination vaccines compared to those who received single-antigen vaccines only [20]. Previous evidence suggested that fears concerning simultaneous vaccination may lead to vaccine avoidance or delay [10,25], and the use of combination vaccines could improve vaccine coverage rates by reducing parental refusal and hesitancy [15,16,26]. With growing concern surrounding the low Hib vaccine coverage rate in China, Hib-containing combination vaccines hold promise for ameliorating this issue. Therefore, the promotion of combination vaccines is suggested as an effective strategy for increasing Hib vaccination rates.

Nevertheless, the cost of trivalent, quadrivalent or pentavalent Hib vaccines surpassed that of the monovalent vaccine significantly, and all of them rely on out-of-pocket payments by caregivers. The procurement prices varied across monovalent, trivalent, quadrivalent and pentavalent doses, with combination vaccines proving notably costlier than monovalent vaccines, and other complementary non-Hib components were offered at no expense within the NIP. In addition to the findings of increased adherence among combination vaccine recipients, the study also explored the influencing factors of caregivers’ choice between different types of Hib-containing vaccines, and observed significant disparities in vaccine uptake and choices. Generally, children who were more socioeconomically deprived were significantly less likely to receive Hib-containing vaccines, i.e., children living in households with lower income had a lower possibility of receiving both monovalent and combination vaccines; caregivers with lower education level were more likely to vaccinate their children against less-expensive monovalent vaccines while less likely to choose combination ones; rural residence and migrant status were negatively associated with combination vaccine receipt. These disparities might be explained by the cost of the vaccines, as nearly all households were required to pay for Hib vaccination by themselves. Previous studies also observed significant socioeconomic disparities in vaccination adherence for DTaP, HPV, MMR, and seasonal influenza vaccines [18]. Thus, policymakers should pay more attention to these potential barriers to immunisation, including income, education and place of residence. Taking appropriate government actions to reduce the financial burden of combination vaccines could help mitigate these disparities.

It is worth noting that when the combination vaccination status was further divided into four subgroups, the positive association between combination vaccine receipt and vaccination completion was no longer evident among those who received AC-Hib only. This can be attributed to the varying vaccination schedules for the AC-Hib vaccine in children of different ages, as children over six months were not recommended to receive three doses. By contrast, the association persisted among those with mixed vaccination, indicating a strong correlation between the completion of the three-dose schedule and mixed combination vaccination. This was observed even though 59% of the mixed vaccination group (127 out of 214) received both monovalent and combination vaccines (**Figure 1**). Therefore, a possible way of facilitating Hib vaccination might be the promotion of at least one-dose vaccination of combination vaccines, which might be easier in practice given the reduced costs. However, due to the limited statistical significance driven by the relatively small sample size in the mixed vaccination group, further exploration is needed to ascertain the effects of mixed vaccination on schedule completion as well as uncover the underlying reasons.

Interestingly, the study found that neither vaccine choice nor completion was associated with parental attitudes and beliefs, or more generally, vaccine hesitancy, although previous studies lacking such assessments of attitudes and beliefs speculated that the failure to receive recommended doses might be attributed to vaccine hesitancy factors [20,27-30]. This may be attributed to the observation that more than four-fifths of caregivers enrolled in this study were vaccine-accepting in terms of importance, safety and efficacy. Previous studies suggested that there may be additional hesitancy specific to combination vaccines and the possibility of overloading the child’s immune system [31], which can be further investigated in future research. Additionally, with the health care facility data on vaccine provision status, we were able to capture vaccine availability in each centre. Our findings demonstrated that Hib vaccination status was significantly related to vaccine availability in frequently visited health care centres. A strong positive relationship was observed between monovalent vaccine receipt and monovalent vaccine supply, as well as between combination vaccine receipt and the supply of both monovalent and combination vaccines. This underscores the importance of proper preparatory measures preparation to facilitate the uptake of Hib-containing vaccines in health care centres.

This study has several limitations. First, about 20% of participants declined to provide their complete vaccination records (19.26%) or the records were illegible owing to blurred pictures (1.35%), which may have led to selection bias. However, the gender and age distribution among children included in this study were similar to the Chinese population eligible for vaccination, and no significant difference was found in terms of children’s age, gender, and vaccine hesitancy indicators when comparing the basic characteristics by vaccination record availability status (Table S1 in the **Online Supplementary Document**), suggesting that the impact of sample selection bias could be minimal. Second, the findings did not necessarily establish a causal relationship between the receipt of combination vaccines and improved adherence owing to the cross-sectional nature of the study. Strict causal inference needs further research. Third, potential bias may be introduced when conducting vaccination-related surveys in vaccination centres since caregivers who decided to reject all vaccines would not be part of the sampling frame, but this bias would be of limited magnitude as the coverage rate of more than one NIP dose is nearly 100% in China in 2019 [24]. Fourth, children who received Hib-containing combination vaccines for only part of the vaccination schedule were grouped as combination vaccine recipients, which might reduce the presumed advantages of combination vaccines in the presence of such mixing.

## CONCLUSIONS

Findings from the current study provide a more comprehensive understanding of the association between receipt of combination vaccines and completion outcomes in China using real-world childhood vaccination records. Strong associations were reported between socioeconomic status and vaccine choices, where more socioeconomically deprived children were less likely to receive Hib-containing vaccination, especially more expensive combination vaccines. Combination vaccine recipients demonstrated a 3-fold increase in the likelihood of three-dose schedule completion compared with monovalent vaccine recipients. As Hib vaccination rate among children remains suboptimal in China, the use of combination vaccines might increase compliance, and appropriate government actions to reduce out-of-pocket burden of immunisation are encouraged to address inequities in vaccine choices.

## Additional material


Online Supplementary Document

## References

[1] He C Liu L Chu Y Perin J Dai L Li X National and subnational all-cause and cause-specific child mortality in China, 1996-2015: a systematic analysis with implications for the Sustainable Development Goals. Lancet Glob Health. 2017;5:e186-97. 10.1016/S2214-109X(16)30334-5 28007477 PMC5250590

[2] WHO Haemophilus influenzae type b (Hib) Vaccination WHO position paper: July 2013-Recommendations. Vaccine. 2013;31:6168-9. 10.1016/j.vaccine.2013.10.045 24156921

[3] Wahl B O’Brien KL Greenbaum A Majumder A Liu L Chu Y Burden of Streptococcus pneumoniae and Haemophilus influenzae type b disease in children in the era of conjugate vaccines: global, regional, and national estimates for 2000-15. Lancet Glob Health. 2018;6:e744-57. 10.1016/S2214-109X(18)30247-X 29903376 PMC6005122

[4] Gupta M Prinja S Kumar R Kaur M Cost-effectiveness of Haemophilus influenzae type b (Hib) vaccine introduction in the universal immunization schedule in Haryana State, India. Health Policy Plan. 2013;28:51-61. 10.1093/heapol/czs025 22407018

[5] Lai X Wahl B Yu W Xu T Zhang H Garcia C National, regional, and provincial disease burden attributed to Streptococcus pneumoniae and Haemophilus influenzae type b in children in China: Modelled estimates for 2010-17. Lancet Reg Health West Pac. 2022;22:100430. 10.1016/j.lanwpc.2022.100430 35308577 PMC8928075

[6] Watt JP Wolfson LJ O’Brien KL Henkle E Deloria-Knoll M McCall N Burden of disease caused by Haemophilus influenzae type b in children younger than 5 years: global estimates. Lancet. 2009;374:903-11. 10.1016/S0140-6736(09)61203-4 19748399

[7] Yu H Yang W Varma JK To save children’s lives, China should adopt an initiative to speed introduction of pneumonia vaccines. Health Aff (Millwood). 2012;31:2545-53. 10.1377/hlthaff.2011.1272 23129686

[8] Marcy SM Pediatric combination vaccines: their impact on patients, providers, managed care organizations, and manufacturers. Am J Manag Care. 2003;9:314-20. 12703675

[9] Standards for pediatric immunization practices. Ad Hoc Working Group for the Development of Standards for Pediatric Immunization Practices. JAMA. 1993;269:1817-22. 8459514

[10] Wallace AS Mantel C Mayers G Mansoor O Gindler JS Hyde TB Experiences with provider and parental attitudes and practices regarding the administration of multiple injections during infant vaccination visits: lessons for vaccine introduction. Vaccine. 2014;32:5301-10. 10.1016/j.vaccine.2014.07.076 25092632

[11] Gust DA Strine TW Maurice E Smith P Yusuf H Wilkinson M Underimmunization among children: effects of vaccine safety concerns on immunization status. Pediatrics. 2004;114:e16-22. 10.1542/peds.114.1.e16 15231968

[12] Marshall GS Happe LE Lunacsek OE Szymanski MD Woods CR Zahn M Use of combination vaccines is associated with improved coverage rates. Pediatr Infect Dis J. 2007;26:496-500. 10.1097/INF.0b013e31805d7f17 17529866

[13] Lyseng-Williamson KA Dhillon S DTPa-HBV-IPV/Hib vaccine (Infanrix hexa™): a guide to its use in infants. Paediatr Drugs. 2012;14:337-43. 22873778 10.2165/11210000-000000000-00000

[14] McCormack PL DTaP-IPV-Hep B-Hib vaccine (Hexaxim®): a review of its use in primary and booster vaccination. Paediatr Drugs. 2013;15:59-70. 10.1007/s40272-013-0007-7 23338932

[15] Maman K Zöllner Y Greco D Duru G Sendyona S Remy V The value of childhood combination vaccines: From beliefs to evidence. Hum Vaccin Immunother. 2015;11:2132-41. 10.1080/21645515.2015.1044180 26075806 PMC4635899

[16] Cohen C von Gottberg A Hib combination vaccines: efficient and effective. Lancet Infect Dis. 2018;18:700-1. 10.1016/S1473-3099(18)30234-2 29752130

[17] Monge S Hahné SJ de Melker HE Sanders EA van der Ende A Knol MJ Effectiveness of the DTPa-HBV-IPV/Hib vaccine against invasive Haemophilus influenzae type b disease in the Netherlands (2003-16): a case-control study. Lancet Infect Dis. 2018;18:749-57. 10.1016/S1473-3099(18)30166-X 29752131

[18] Loiacono MM Pool V van Aalst R DTaP combination vaccine use and adherence: A retrospective cohort study. Vaccine. 2021;39:1064-71. 10.1016/j.vaccine.2021.01.009 33483215

[19] Happe LE Lunacsek OE Kruzikas DT Marshall GS Impact of a pentavalent combination vaccine on immunization timeliness in a state Medicaid population. Pediatr Infect Dis J. 2009;28:98-101. 10.1097/INF.0b013e318187d047 19148039

[20] Kurosky SK Davis KL Krishnarajah G Effect of combination vaccines on completion and compliance of childhood vaccinations in the United States. Hum Vaccin Immunother. 2017;13:2494-502. 10.1080/21645515.2017.1362515 28881166 PMC5703402

[21] Happe LE Lunacsek OE Marshall GS Lewis T Spencer S Combination vaccine use and vaccination quality in a managed care population. Am J Manag Care. 2007;13:506-12. 17803364

[22] Lai X Rong H Ma X Hou Z Li S Jing R Willingness to Pay for Seasonal Influenza Vaccination among Children, Chronic Disease Patients, and the Elderly in China: A National Cross-Sectional Survey. Vaccines (Basel). 2020;8:405. 10.3390/vaccines8030405 32707831 PMC7563663

[23] Li X Lu J Hu S Cheng KK De Maeseneer J Meng Q The primary health-care system in China. Lancet. 2017;390:2584-94. 10.1016/S0140-6736(17)33109-4 29231837

[24] GBD 2020, Release 1, Vaccine Coverage Collaborators. Measuring routine childhood vaccination coverage in 204 countries and territories, 1980-2019: a systematic analysis for the Global Burden of Disease Study 2020, Release 1. Lancet. 2021;398:503-21. 10.1016/S0140-6736(21)00984-3 34273291 PMC8358924

[25] Gust DA Darling N Kennedy A Schwartz B Parents with doubts about vaccines: which vaccines and reasons why. Pediatrics. 2008;122:718-25. 10.1542/peds.2007-0538 18829793

[26] Centers for Disease Control and Prevention General recommendations on immunization—recommendations of the Advisory Committee on Immunization Practices (ACIP). MMWR Recomm Rep. 2011;60:1-64. 21293327

[27] Salmon DA Dudley MZ Glanz JM Omer SB Vaccine hesitancy: Causes, consequences, and a call to action. Vaccine. 2015;33 Suppl 4:D66-71. 10.1016/j.vaccine.2015.09.035 26615171

[28] Nadeau JA Bednarczyk RA Masawi MR Meldrum MD Santilli L Zansky SM Vaccinating my way–use of alternative vaccination schedules in New York State. J Pediatr. 2015;166:151-6. 10.1016/j.jpeds.2014.09.013 25444525

[29] McCauley MM Kennedy A Basket M Sheedy K Exploring the choice to refuse or delay vaccines: a national survey of parents of 6- through 23-month-olds. Acad Pediatr. 2012;12:375-83. 10.1016/j.acap.2012.06.007 22921495

[30] Smith PJ Humiston SG Marcuse EK Zhao Z Dorell CG Howes C Parental delay or refusal of vaccine doses, childhood vaccination coverage at 24 months of age, and the Health Belief Model. Public Health Rep. 2011;126 Suppl 2:135-46. 10.1177/00333549111260S215 21812176 PMC3113438

[31] Gidengil C Lieu TA Payne K Rusinak D Messonnier M Prosser LA Parental and societal values for the risks and benefits of childhood combination vaccines. Vaccine. 2012;30:3445-52. 10.1016/j.vaccine.2012.03.022 22449423 PMC8654055

