# Supplementary material for: Better adherence to childhood Haemophilus influenzae type b vaccination with combination vaccines compared to single-antigen vaccines: Evidence from China
Source: J Glob Health. 2023 Aug 25;13:04080. doi: 10.7189/jogh.13.04080 (PMC10451103; doi:10.7189/jogh.13.04080)
Supplement: Online Supplementary Document [file jogh-13-04080-s001.pdf]

Online supplementary document for

Better adherence to childhood Haemophilus influenzae type b vaccination with combination vaccines compared to single-antigen vaccines: evidence from China

Description of the multistage sampling cluster method and study sites: In this study, children aged 6-59 months were enrolled through a **multistage cluster sampling method**:

First, seven provinces (Jilin, Henan, Jiangxi, Shandong, Guangdong, Gansu, and Yunnan) and three provincial-level cities (Beijing, Shanghai, and Chongqing) were selected based on China's Division of Central and Local Financial Governance and Expenditure Responsibilities in the Healthcare Sector (http://www.gov.cn/zhengce/content/2018-08/13/content_5313489.htm), which stratifies the 31 provinces/provincial-level cities into five layers according to the socio-economic development and abilities of local governments. In terms of location and socioeconomic development, ten provinces/provincial-level cities (3, 3, 1, 1, and 2 in each layer) were chosen to represent different regions of China, with their ranks of 2018 per capita GDP (e.g., 1/31) recorded in the following map figure.

Second, a capital city and a non-capital city were selected in each province. For provincial-level cities, an economically developed district and a less-developed district were selected accordingly.

Third, two subdistricts/counties were chosen in each city or district, among which one was comparatively more developed and the other was less developed in the city or district.

Fourth, in each subdistrict/county, three to four communities and the corresponding vaccination centers (based in community health centers) were sampled to represent low (below median), median and high (above median) social-economic strata.

Fifth, in each vaccination center, guardians of all children aged 6-59 months visiting

the sampled vaccination centers on a given day during the survey period were invited to participate in the survey.

The minimum sample size was calculated under the assumption that the predicted proportion of delayed doses is 50%. With an allowable error of 5%, the sample size was set to 384 in each province and 3,840 as a total across ten provinces. In our survey, oversampling was used to consider the potential response rate and integrity of data collected. Actually, 6,668 children were recruited in the survey. Among them, the guardians of 5,384 (80.74%) children agreed to provide their vaccination records, and the records of 5,294 (79.39%) were legible and complete with snapshots of every page.

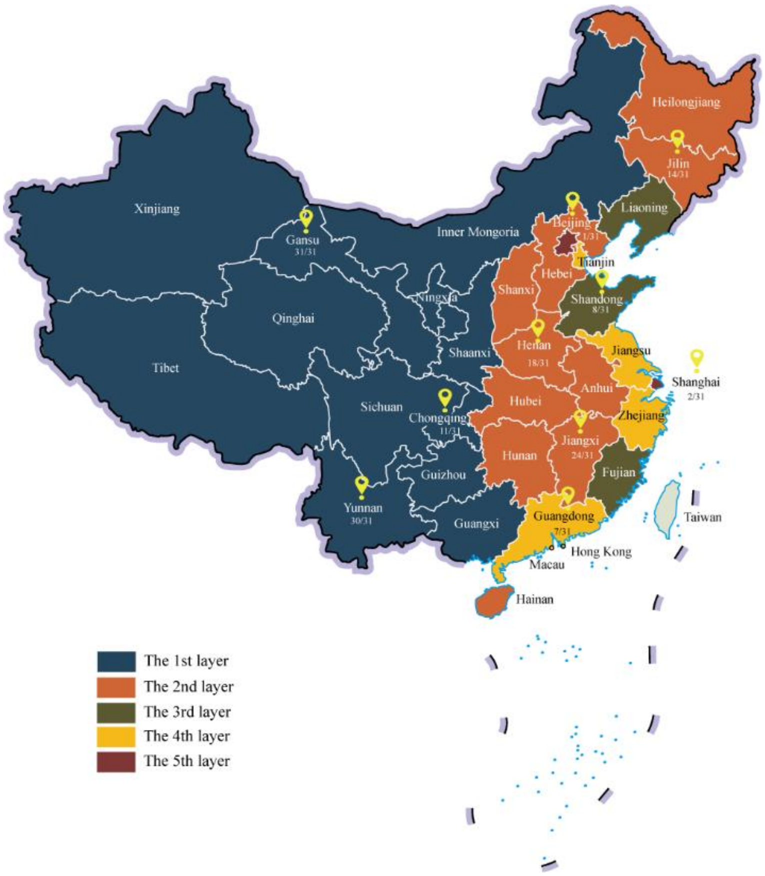

Figure S1. Ten sampled provinces or provincial-level cities in China for the survey. Ranks of 2018 per capita GDP by province (e.g., 1/31) are marked in the figure.

Table S1. Basic characteristics of respondents by vaccination record availability status.

	Overall		Record available		Record unavailable		*p-value**
	N	Column %	N	Column %	N	Column %	
**Total**	6,668	100.00	5,294	100.00	1,374	100.00	
Respondent's age (years)							< 0.001
<30	2049	30.73	1637	30.92	412	29.99	
30-39	3039	45.58	2457	46.41	582	42.36	
40-49	569	8.53	458	8.65	111	8.08	
≥50	1011	15.16	742	14.02	269	19.58	
Respondent's relationship with the child							< 0.001
Mather	4405	66.06	3534	66.75	871	63.39	
Father	1122	16.83	907	17.13	215	15.65	
Grandparent	1141	17.11	853	16.11	288	20.96	
Respondent's education level							0.651
Elementary school and below	674	10.11	521	9.84	153	11.14	
Middle school	1710	25.64	1355	25.60	355	25.84	
Senior high school/Technical school	1503	22.54	1194	22.55	309	22.49	
College/Associate degree	1250	18.75	997	18.83	253	18.41	
Bachelor's degree and above	1531	22.96	1227	23.18	304	22.13	
Belonging to minority groups							0.041
Yes	371	5.56	310	5.86	61	4.44	
No	6297	94.44	4984	94.14	1313	95.56	
Child's age (years)							0.091
<1	1768	26.51	1439	27.18	329	23.94	

1-2	1959	29.38	1547	29.22	412	29.99	
2-3	1241	18.61	981	18.53	260	18.92	
3-5	1700	25.49	1327	25.07	373	27.15	
Child's gender							0.235
Female	3171	47.56	2498	47.19	673	48.98	
Male	3497	52.44	2796	52.81	701	51.02	
Consider vaccine as important							0.456
Yes	6493	97.38	5159	97.45	1334	97.09	
No	175	2.62	135	2.55	40	2.91	
Consider vaccine as safe							0.367
Yes	5376	80.62	4280	80.85	1096	79.77	
No	1292	19.38	1014	19.15	278	20.23	
Consider vaccine as effective							0.095
Yes	5315	79.71	4242	80.13	1073	78.09	
No	1353	20.29	1052	19.87	301	21.91	

* P-values from Chi-square test.

Hib, *Haemophilus influenzae type b*.
